# Supplementary material for: Thermal window of exercise performance of the ecosystem engineer Lanice conchilega
Source: Biol Open. 2026 Jan 23;15(1):bio062398. doi: 10.1242/bio.062398 (PMC12869513; doi:10.1242/bio.062398)
Supplement: Supplementary information [file biolopen-15-062398-s1.pdf]

Table S1. Number of aquaria that were used across experimental days and temperatures. Each aquarium contained two *Lanice conchilega* individuals whose performance was averaged. n = replicates

		Experimental temperature (°C)									
		5	7	9	11	13	15	17	19	21	23
Experimental day	1			2	2			2	2		
	2				2	2				2	2
	3			2	2		2	2			
	4		1		1		2		2		
	5		3		2					2	2
	6	3		2	2	2					
	7	2	1		2	2					
	8				2		2	1	2		
Total n		5	5	6	15	6	6	5	6	4	4

Table S2. Model performance and thermal performance estimates. ΔAICc = difference in Akaike Information Criterion (AICc) between the best and the specified model as a measure of relative model fit. T_{opt} = thermal optimum (°C), R_{max} = performance maximum (cm), T_{min} = thermal minimum (°C), T_{max} = thermal maximum (°C). Thermal performance estimates are stated as mean with 95% confidence intervals in parentheses.

Model	Model weight	ΔAICc	T_{opt} (95% CI) (°C)	R_{max} (95% CI) (cm)	T_{min}(95% CI) (°C)	T_{max} (95% CI) (°C)
Gaussian	0.624	0	12.4 (11.9–13.1)	8.9 (7.3–11.0)	3.6 (1.8–6.3)	21.4 (19.6–22.9)
Oneill	0.188	3	12.5 (11.5–13.1)	8.9 (7.5–10.8)	-32.6 (-44.3– -1.0)	53.2 (40.8–66.4)
Weibull	0.171	3	13.2 (11.2–13.3)	8.8 (7.4–10.5)	4.3 (0.3– 5.5)	21.2 (19.4–58.4)
Pawar	0.017	8	12.5 (12.1–13.8)	8.8 (7.4–10.8)	-4.6 (-8.8– -1.9)	21.5 (18.6–24.1)

Biology Open • Supplementary information
